# Ultrasonication-based rapid amplification of α-synuclein aggregates in cerebrospinal fluid

**DOI:** 10.1038/s41598-019-42399-0

**Published:** 2019-04-12

**Authors:** Keita Kakuda, Kensuke Ikenaka, Katsuya Araki, Masatomo So, César Aguirre, Yuta Kajiyama, Kuni Konaka, Kentaro Noi, Kousuke Baba, Hiroshi Tsuda, Seiichi Nagano, Takuma Ohmichi, Yoshitaka Nagai, Takahiko Tokuda, Omar M. A. El-Agnaf, Hirotsugu Ogi, Yuji Goto, Hideki Mochizuki

**Affiliations:** 1 Department of Neurology, Graduate School of Medicine, Osaka University, Yamadaoka 2-2, Suita, Osaka 565-0871 Japan; 2 Institute for Protein Research, Osaka University, Yamadaoka 3-2, Suita, Osaka 565-0871 Japan; 3 Department of Precision Science and Technology, Graduate School of Engineering, Osaka University, Yamadaoka 2-1, Suita, Osaka 565-0871 Japan; 4 Department of Neurology, Kyoto Prefectural University of Medicine, 465 Kajii-cho, Kamikyo-ku, Kyoto 602-8566 Japan; 5 Department of Neurotherapeutics, Graduate school of Medicine, Osaka University, Yamadaoka 2-2, Suita, Osaka 565-0871 Japan; 6 Department of Molecular Pathobiology of Brain Diseases, Kyoto Prefectural University of Medicine, 465 Kajii-cho, Kamikyo-ku, Kyoto 602-8566 Japan; 7 Life Sciences Division, College of Science and Engineering, Hamad Bin Khalifa University (HBKU), Education City, Qatar

## Abstract

α-Synuclein aggregates, a key hallmark of the pathogenesis of Parkinson’s disease, can be amplified by using their seeding activity, and the evaluation of the seeding activity of cerebrospinal fluid (CSF) is reportedly useful for diagnosis. However, conventional shaking-based assays are time-consuming procedures, and the clinical significance of the diversity of seeding activity among patients remains to be clarified. Previously, we reported a high-throughput ultrasonication-induced amyloid fibrillation assay. Here, we adapted this assay to amplify and detect α-synuclein aggregates from CSF, and investigated the correlation between seeding activity and clinical indicators. We confirmed that this assay could detect α-synuclein aggregates prepared *in vitro* and also aggregates released from cultured cells. The seeding activity of CSF correlated with the levels of α-synuclein oligomers measured by an enzyme-linked immunosorbent assay. Moreover, the seeding activity of CSF from patients with Parkinson’s disease was higher than that of control patients. Notably, the lag time of patients with Parkinson’s disease was significantly correlated with the MIBG heart-to-mediastinum ratio. These findings showed that our ultrasonication-based assay can rapidly amplify misfolded α-synuclein and can evaluate the seeding activity of CSF.

## Introduction

Parkinson’s disease (PD) is the second most common neurodegenerative disease and is characterized by motor symptoms, such as bradykinesia, rigidity, tremor, and gait disturbance, mainly due to the loss of dopaminergic neurons in the substantia nigra pars compacta. Current treatments for PD are restricted to dopamine replacement therapy, which only improves the motor symptoms without affecting disease progression^1^, and no disease-modifying therapies have been developed.

The etiology of PD is still not clear, but studies have suggested that the aggregation and propagation of misfolded α-synuclein play key roles in disease initiation and progression^2–6^. Therefore, one of the most promising strategies to develop disease-modifying therapies is to prevent the accumulation of misfolded α-synuclein aggregates, e.g., by antibody therapy^7,8^. For the development of these therapies, it is important to provide an accurate diagnosis at a very early stage of the disease and it is necessary to develop accurate biomarkers that can be used to assess the degree of the accumulation of α-synuclein aggregates in the brain of patients to evaluate the efficacy of those treatments.

An enzyme-linked immunosorbent assay (ELISA) has been developed to assess the levels of α-synuclein, as a candidate biomarker, and several recent papers have demonstrated changes of α-synuclein levels in the cerebrospinal fluid (CSF) of patients with PD. Total α-synuclein levels were found to be decreased in the CSF of patients with PD compared to normal controls^9–12^, while the levels of oligomeric α-synuclein species were increased^13–17^. However, the sensitivity and specificity of the ELISA system are still not sufficient for clinical use by itself^18^.

Recently, some groups have applied the Protein Misfolded Cyclic Amplification (PMCA) or Real-Time Quaking-Induced Conversion (RT-QuIC) assays, which were established initially for the detection of abnormal prion protein in Creutzfeldt-Jakob disease, to the amplification of misfolded α-synuclein aggregates from brain lysates or CSF samples of patients with PD. These assays were shown to amplify specifically α-synuclein aggregates from patients and could be used to evaluate the amount of aggregates by monitoring amplification kinetics. Using this technique, PD patients could be separated from controls^19–25^. These reports suggest that the detection of α-synuclein aggregates in CSF by specific amplification may offer a good opportunity for the biochemical diagnosis of the disease.

In order to determine further the clinical and pathological relevance of α-synuclein aggregates in CSF, additional studies are required to compare their kinetics with clinical and imaging parameters. To clarify this, we conducted a cross-sectional study to analyze the seeding activity of CSF with detailed clinical information and radiographic examinations in a single-center prospective cohort of patients with PD.

In this study, we employed our novel system, HANdai Amyloid Burst Inducer (HANABI), which induces efficient amyloid fibril formation by automated sonication and an incubation cycle with real-time monitoring of a fluorescent signal^26,27^. The greatest advantage of using this system is that it dramatically shortens the time to perform the assay from the approximately 10 days for the shaking-based assays (PMCA and RT-QuIC) to only several hours^26,27^.

Using this system, we could detect seeding-competent α-synuclein aggregates in CSF from patients and found a difference in the seeding activity of CSF from patients with PD compared to controls. We also demonstrated a correlation between the seeding activity of CSF and detailed clinical parameters, including disease severity and radiographic examinations.

## Results

### Ability of the HANABI assay to detect α-synuclein pre-formed fibrils (PFFs) and released aggregates from cells *in vitro*

**Figure 1:**
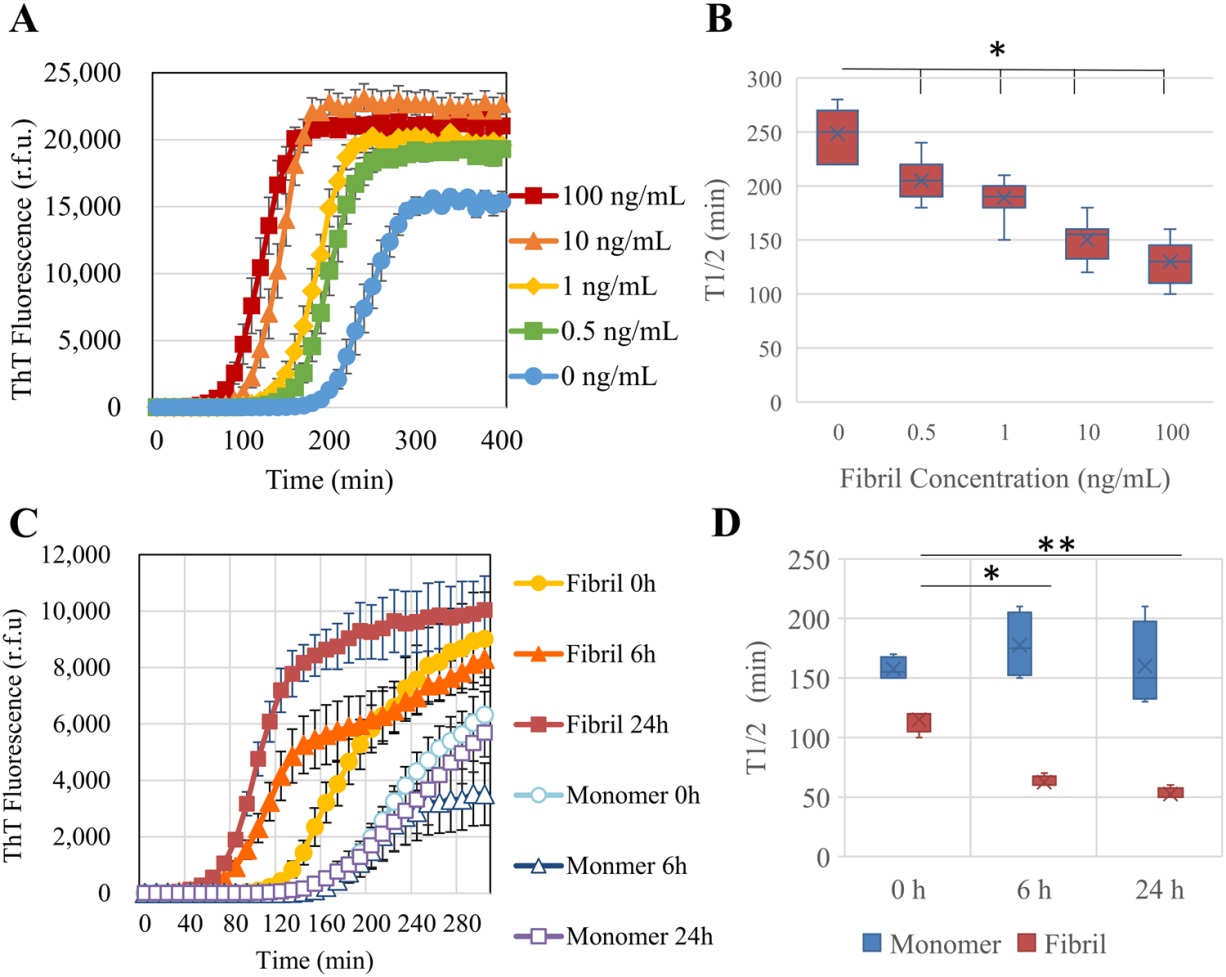
Detection of α-synuclein oligomers/fibrils with the HANABI assay. (**A**) Average time curve of ThT fluorescence with *in vitro* PFFs. The increase of ThT fluorescence was accelerated with the increase of PFF concentration. (**B**) The time to reach half maximum fluorescence (T1/2) was shortened with PFFs (mean ± standard deviation = 248.6 ± 23.4, 205.0 ± 18.4, 189.0 ± 19.2, 150.0 ± 17.0, and 130.0 ± 20.6 min for 0, 0.5, 1.0, 10.0, and 100 ng/mL PFFs, respectively, *p < 0.05, one-way ANOVA with post hoc Tukey HSD test). (**C**) Average time curve of the ThT assay with media from α-synuclein-overexpressing cells seeded with PFFs or controls (seeded with α-synuclein monomers). The media of the cells seeded with PFFs showed a rapid increase of ThT fluorescence, and these effects were increased in a time-dependent manner after seeding. (**D**) Media collected after a longer period of time after seeding showed shorter lag times when the cells were seeded with PFFs (115 ± 10, 62.5 ± 5.0, and 52.5 ± 5.0 min for 0, 6, and 24 h, respectively, *n* = 3, *p < 0.05 and **p < 0.01, two-way ANOVA with post hoc Bonferroni test), but not when they were seeded with monomers.

To study the ability of the HANABI assay to detect small amounts of α-synuclein aggregates, we tested several models mimicking the CSF of patients with PD. First, we used PFFs made from purified recombinant human wild-type (WT) full-length α-synuclein by intermittent sonication. Seeded by PFFs, amyloid formation was accelerated in a dose-dependent manner, which was evaluated by monitoring thioflavin T (ThT) fluorescence (Fig. 1A). We quantified the speed of fibril formation by calculating the average time to reach half maximum fluorescence (T1/2). The T1/2 values of the measurements with PFFs were significantly shorter than without PFFs. On the basis of this PFF study, we found that the HANABI assay could detect as little as 0.5 ng/mL PFFs (Fig. 1B).

In order to investigate whether the HANABI assay could detect α-synuclein oligomers released from neurons into the extra-cellular space, we analyzed the culture media of neuronal cells in which α-synuclein aggregation was induced. We cultured SH-SY5Y cells overexpressing α-synuclein, and induced aggregation by transfecting the cells with PFFs. At 24 h after PFF transfection, the cells were washed carefully with phosphate-buffered saline (PBS) to remove extracellular PFFs, and we collected the medium sequentially at 0, 6, and 24 h and cell lysate at 24 h. Western blot analysis of the cell lysates confirmed the increase of α-synuclein aggregates in the cells induced by PFFs, both in the soluble and insoluble fractions (Supplementary Figs S1–S3). The extracellular medium of cells with PFF-induced aggregation showed an incubation time-dependent acceleration of the seeding reaction (Fig. 1C). The T1/2 values of medium collected from aggregation-induced cells after longer incubation times were significantly shortened in a time-dependent manner, suggesting the increased release of α-synuclein aggregates from the cells. Thus, the HANABI assay was able to reflect the amount of α-synuclein aggregates released from cultured neuronal cells as the acceleration of the seeding reaction.

### The HANABI assay reflects α-synuclein oligomers in CSF of patients with PD measured by an ELISA

**Figure 2:**
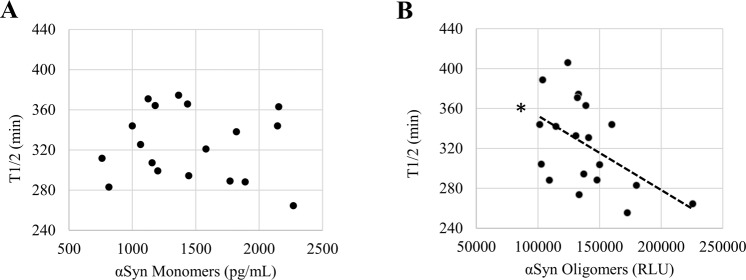
The HANABI assay reflects α-synuclein oligomers in CSF from patients with PD measured by ELISA. (**A**,**B**) Dot plot of T1/2 and the levels of α-synuclein monomers and oligomers in CSF measured by ELISA. T1/2 of CSF from patients with PD was correlated with the levels of CSF oligomers (Pearson correlation test, r = −0.549, p = 0.018), but not correlated with the CSF concentrations of α-synuclein monomers.

To determine whether the seeding activity detected by the HANABI assay reflects the elevation of α-synuclein aggregates in CSF, we applied this assay to CSF from patients with PD and compared the results with an ELISA for CSF oligomers. Importantly, the results from HANABI, i.e., T1/2, were correlated with the amount of CSF oligomers of α-synuclein analyzed by the ELISA, but not with monomers (Fig. 2A,B); CSF with higher amounts of α-synuclein oligomers measured by the ELISA also showed higher seeding activity in the HANABI assay. In addition, we analyzed whether the kinetics of the HANABI assay correlated with the oligomerization activity of CSF. The increase of oligomers in an α-synuclein monomer solution incubated with CSF was evaluated over time by western blot analysis (Supplementary Fig. S4A,B). The oligomerization activity of CSF from each patient, calculated by the ratio of oligomers after 4-day incubation/before incubation, was significantly correlated with T1/2 and also with α-synuclein oligomers in CSF measured by ELISA (Supplementary Fig. S4C,D). These results strongly suggest that the HANABI assay is a useful tool to analyze small concentrations of α-synuclein aggregates in CSF from patients with PD. Moreover, all measurements of the HANABI assay were finished within 10 h, which is a highly reduced time compared to the conventional quaking-based assays that take several days.

### Difference between PD and control patients in the HANABI assay

**Figure 3:**
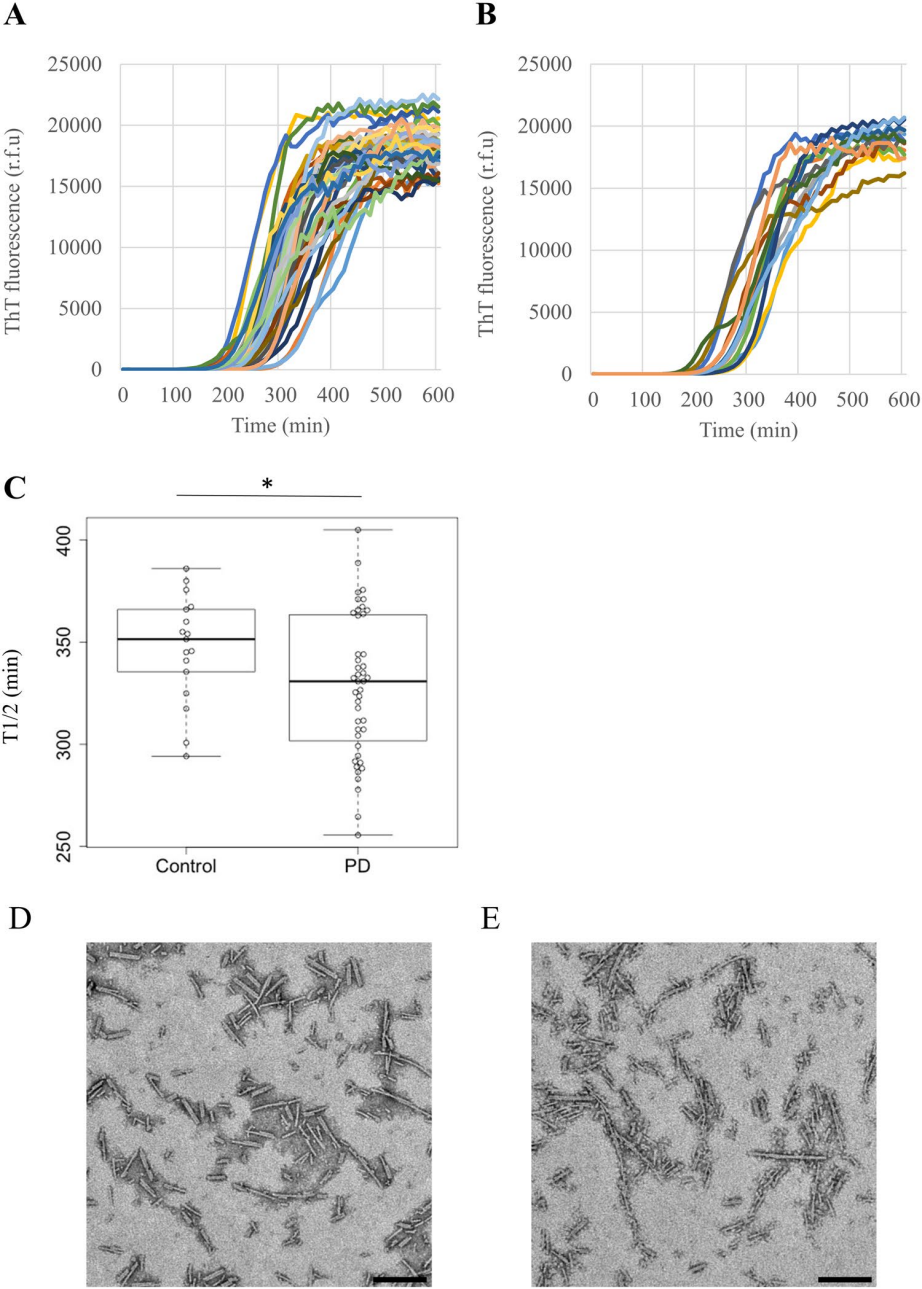
HANABI kinetics of PD and control patients. (**A**) Average time curve of patients with PD and (**B**) control patients. (**C**) Box plot and beeswarm plot of T1/2 of patients with PD (PD) and control patients. T1/2 of patients with PD was shorter than that of control patients (328.5 ± 34.9 vs. 348.3 ± 27.1 min, respectively, p = 0.046, unpaired t-test). (**D**,**E**) TEM images of representative fibrils made by the HANABI assay with CSF from patients with PD (**D**) and control patients (**E**). Scale bar, 200 nm.

In order to investigate the clinical significance of the results of the HANABI assay, we compared patients with PD and disease controls. Among the 261 patients who participated in our PD prospective observational study from July 2016 to June 2017, 69 received a CSF examination, and 44 of those were diagnosed as clinically established PD or probable PD based on the criteria of the Movement Disorder Society (MDS)^28^. As a disease control, we employed 17 patients without a neurodegenerative or neuroinflammatory disease who received a CSF examination during the same period, including patients with psychiatric movement disorders without evidence of an organic cause (*n* = 6), normal pressure hydrocephalus (*n* = 3), myopathies (*n* = 3), spondylosis (*n* = 2), dystonia (*n* = 1), myasthenia gravis (*n* = 1), and orthostatic hypotension (*n* = 1). The clinical data of the patients are shown in Table 1. All patients with PD were admitted to hospital for clinical diagnosis or treatment and received detailed examinations of motor and non-motor functions. Since we collect samples and clinical data at the point of diagnosis, our PD group contained relatively early-stage patients (Table 1). There were no significant differences in age, sex, and CSF protein concentration between the PD and control groups. The average T1/2 value of the patients with PD was shorter than that of the control patients (Fig. 3A–C); however, there were overlaps between both groups. The fibrils made by the HANABI assay using CSF from both groups were examined with transmission electron microscopy (TEM). Both groups contained fragmented filamentous fibrils; however, there was no consistent difference in their structures between the groups (Fig. 3D,E). The T1/2 values of the PD group seemed heterogeneous with a bimodal-like distribution. In order to understand the clinical features of those patients, we performed further analysis between the HANABI data and detailed clinical and radiological data.

**Table 1:** Clinical features of PD and control patients.

	PD (*n* = 44)	Controls (*n* = 17)	P-value
Age (years)	66 ± 11	66 ± 14	0.95
CSF protein (mg/dL)	45 ± 16	43 ± 14	0.73
Disease duration (years)	4.9 ± 4.1		
Hoehn-Yahr stage	2.6 ± 0.9		
MDS-UPDRS part III	27.2 ± 14.9		
Medication; levodopa (mg)	181.8 ± 231.3		
Medication; levodopa equivalent dose (mg)	306.0 ± 419.9		
Mini-Mental State Examination	27.9 ± 2.7		
10-m timed up and go test (s)	12.4 ± 7.5 (*n* = 41)		
MIBG H/M ratio; delayed phase	2.0 ± 0.8 (*n* = 33)		
DAT SPECT specific binding ratio (lower ratio of each hemisphere)	4.7 ± 2.2 (*n* = 34)		

Note: Numerical data are given as mean ± standard deviation. *n* stands for the number of subjects who received each examination.

### Correlations between the CSF-HANABI assay and clinical information in patients with PD

**Figure 4:**
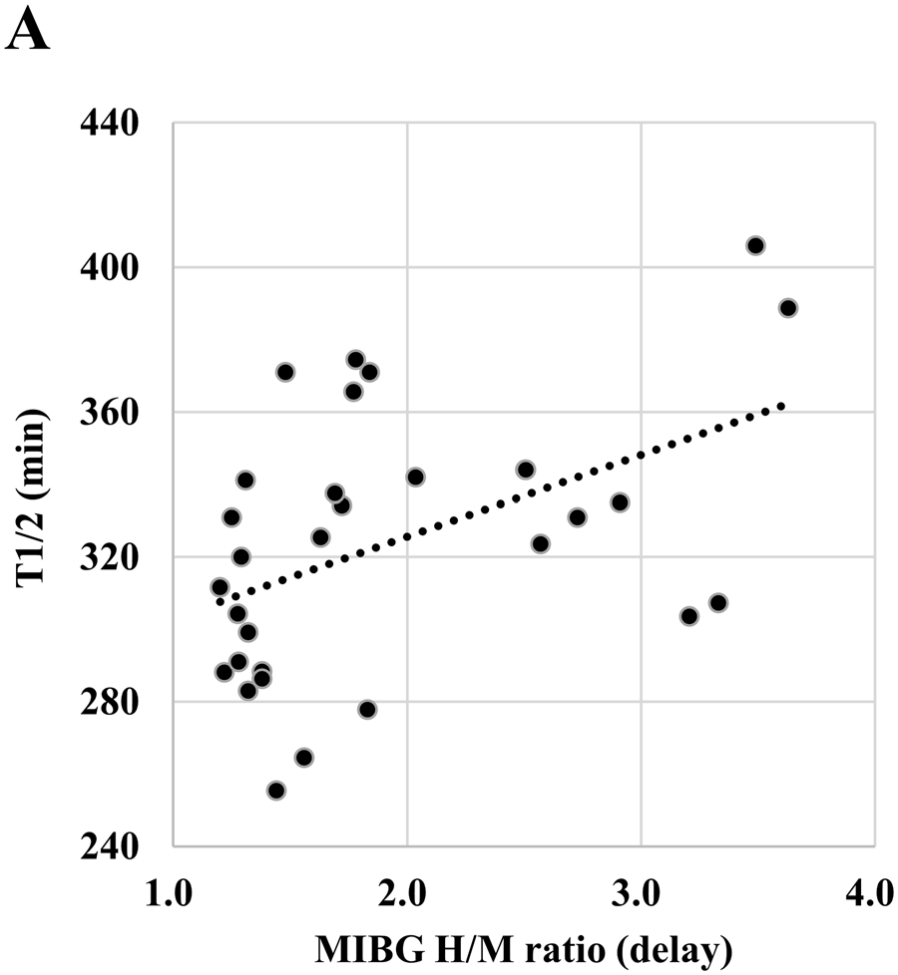
Correlation between HANABI kinetics and the MIBG H/M ratio. Dot plot of T1/2 and the MIBG H/M ratio of patients with PD. There was a positive correlation between both parameters (ρ = 0.405, p = 0.019, Spearman correlation test).

To investigate whether the results from the HANABI assay correlated to the disease severity and/or pathology of patients with PD, we analyzed the HANABI results with clinical parameters. Multiple linear regression analysis was performed to predict T1/2 based on age, disease duration, Hoehn-Yahr severity stage, ^123^I-meta-iodobenzylguanidine (MIBG) heart-to-mediastinum (H/M) ratio, and total protein level in the CSF. Among them, only the MIBG H/M ratio was an independent predictor of T1/2 (Table 2). Namely, CSF from patients with a lower MIBG H/M ratio showed stronger seeding activity in the HANABI assay, implying high levels of α-synuclein aggregates in their CSF (Fig. 4).

**Table 2:** Multiple linear regression to predict CSF seeding activity (T1/2) based on age, disease duration, Hoehn-Yahr severity stage, and MIBG H/M ratio.

	95% confidence interval of standardized coefficient	P-value
Age	−1.91–0.73	0.366
Disease duration	−4.56–3.21	0.725
Hoehn Yahr stage	−0.28–33.2	0.054
MIBG H/M ratio; delay phase	2.13–38.1	0.030*
Total protein levels of CSF	−0.37–1.55	0.220

## Discussion

In this study, we described a technique with the HANABI device that can be used to amplify seeding-competent α-synuclein aggregates by inducing fibril formation with ultrasonication. Our results showed that the HANABI assay could detect PFFs dissolved in artificial CSF, released aggregates from α-synuclein aggregation-induced cells, and α-synuclein aggregates in the CSF of patients with PD. In recent years, great attention has been paid to the specific amplification of α-synuclein aggregates from the brain or CSF of patients with PD by the PMCA^20,25^ and RT-QuIC assays^19,21–23^, which were established initially to detect misfolded prion protein in patients with Creutzfeldt-Jakob disease. These studies have shed light on the evaluation of misfolded α-synuclein aggregates in the central nervous system of living patients.

The conventional PMCA assay requires the manual processing of sonication-incubation cycles and measurement of ThT fluorescence, and it has difficulties with the real-time monitoring of fibril growth. One device designed for the RT-QuIC assay uses automated cycles with vigorous shaking instead of ultrasonication for amplification. By overcoming the technical difficulties associated with realizing an automated ultrasonication-based system that could process multiple samples at once, we recently developed HANABI, a machine composed of a three-directional ultrasonicator in a water bath that was focused near the surface of the water, a mechanical arm to manipulate a microplate containing samples, and a 96-well microplate reader^26,27^. This assay uses the advantages of both existing techniques; ultrasonication amplifies α-synuclein seeds more rapidly than orbital shaking, resulting in a dramatic shortening of the time necessary to perform the assay from several days to several hours, and automated cycles with a 96-well plate to measure a large number of samples simultaneously.

This is the first report to analyze the correlation between the seeding activity of α-synuclein in CSF from patients with PD and comprehensive clinical parameters including age, disease severity, disease duration, and radiographic examinations. Most strikingly, the HANABI results were significantly correlated with the MIBG H/M ratio. Reduced cardiac uptake of MIBG has been accepted as one of the most specific radiological features of PD, dementia with Lewy bodies, and synuclein-related familial forms of PD (PARK1 and PARK4), all of which share Lewy body pathology in the brain^29–32^. In Japan, since the usefulness of MIBG has been studied exhaustively, it has become a general examination for the diagnosis of PD, and the latest MDS clinical diagnostic criteria for PD adopted reduced MIBG uptake as a supportive criterion^28^. The significance of MIBG has been reinforced by pathological findings that cardiac sympathetic nerves are dramatically decreased in patients with PD or dementia with Lewy bodies with the preceding accumulation of Lewy bodies in their distal axons^33,34^. A decrease in the uptake of MIBG and Lewy body pathology in cardiac sympathetic nerves were even observed in patients with incidental Lewy body disease (ILBD), which is considered to represent the prodromal period of PD, and in patients with REM sleep behavior disorder (RBD), who are at a high risk of developing PD^35–38^. Several cross-sectional studies have shown a correlation between low MIBG uptake and various non-motor symptoms of PD, including hyposmia, RBD, cognitive deficits, and visual hallucinations^39–42^. Recent longitudinal studies of patients with PD showed that MIBG uptake declined chronologically, and severely reduced MIBG uptake at baseline was correlated with the faster progression of motor dysfunction and increased likelihood of developing dementia^43,44^. On the basis of these observations, low MIBG uptake is considered to be related to the wider extension of neurodegeneration with Lewy body pathology. Therefore, our data, showing a correlation between the HANABI assay and MIBG uptake, suggest that the seeding activity of CSF from patients with PD could reflect the progression of Lewy body pathology. Further studies are needed to prove this hypothesis, and we are currently attempting to confirm this pathologically.

Some limitations should be noted in this study. First, the lag times of fibril formation in patients with PD were significantly shorter than those of controls in the HANABI assay; however, it could not divide both groups clearly because of an overlap in their times. This overlap might partly be due to the following facts. Since the sonication power of HANABI is strong and not well-controlled, it induces not only secondary nucleation from existent fibrils but also primary nucleation from monomers. This might have resulted in a reduction of specificity in patients without larger amounts of CSF aggregates. On this point, we are developing a next-generation HANABI machine in which sonication frequency and strength can be controlled in combination with a monitoring and feed-back sonication system in each well, which will realize appropriate and homogeneous sonication that is focused on the secondary amplification of seeds in CSF without primary nucleation from monomers. Another explanation for this overlap may partly be due to the accuracy of clinical diagnosis. In the PD group, patients with other forms of parkinsonism lacking Lewy body pathology, such as progressive supranuclear palsy or corticobasal degeneration, might be included, since our cohort mainly targeted the early stage of the disease, and other forms of parkinsonism sometimes mimic PD at this stage. Indeed, it has been reported that 5–25% of patients diagnosed with PD during their lifetime have a different diagnosis after autopsy^45–48^. On the contrary, some patients in the control group might have Lewy body pathology without clinical symptoms, because ILBD has been reported to account for 10–12% of the clinically healthy elderly population^49–51^. The second limitation is that although the results of the HANABI assay correlated with the number of α-synuclein oligomers measured by ELISA, it gave a wide range of T1/2 values for individual concentrations of oligomers. This might have happened because of the presence of factors in CSF that can influence the aggregation of α-synuclein. Indeed, in this study, the lag times of the aggregation reaction using CSF samples were longer than those of the *in vitro* assay without PFFs, which is consistent with an inhibitory effect of CSF on this reaction^19^. The diversity of those inhibitory factors may account for the wide distribution of T1/2. To the best of our knowledge, there are no reports describing specific factors in CSF that can inhibit α-synuclein aggregation; therefore, further studies are needed to elucidate this.

In conclusion, our results showed that the sonication-based HANABI assay is a very rapid and useful tool to detect α-synuclein aggregates. The seeding activity of CSF was correlated with reduced MIBG uptake, suggesting the possibility that it reflects Lewy body pathology. Further improvement of the control of ultrasonication is needed for the clinical use of the seeding activity of CSF as a biomarker for PD.

## Methods

### Purification of recombinant α-synuclein

Human WT α-synuclein was purified from *Escherichia coli* as described previously^52^. Briefly, a plasmid containing WT human α-synuclein was expressed in *E. coli* BL21 (DE3) (Novagen, Merck, San Diego, CA, USA). The cells were suspended in a purification buffer, disrupted by sonication, and centrifuged. Streptomycin sulfate (final 2.5% [w/w]) was added to the supernatant and centrifuged again. The supernatant was heated at 90 °C in a water bath and centrifuged. The supernatant was precipitated by the addition of solid ammonium sulfate to 70% saturation, centrifuged, dialyzed overnight, and applied onto a Resource-Q column (GE Healthcare, Little Chalfont, UK) with 50 mM Tris-HCl buffer (pH 7.5) containing 0.1 mM dithiothreitol and 0.1 mM phenylmethylsulfonyl fluoride as a running buffer, and eluted with a linear gradient of 0–1 M NaCl. α-Synuclein-enriched fractions (as determined by sodium dodecyl sulfate-polyacrylamide gel electrophoresis [SDS-PAGE]/Coomassie blue staining) were pooled and further purified by size exclusion chromatography using a Superdex 200 10/300 GL column (GE Healthcare, Little Chalfont, UK) equilibrated with 50 mM Tris-HCl (pH 7.5), 150 mM NaCl. Pure fractions were combined and dialyzed against deionized water at 4 °C. Protein purity was confirmed to be greater than 95% by SDS-PAGE and matrix-assisted laser desorption/ionization mass spectrometry. The sample solution was flash-frozen in liquid nitrogen and lyophilized.

### Preparation of PFFs

Recombinant α-synuclein monomers diluted in buffer (150 mM NaCl, 50 mM Tris-HCl, pH 7.4) were sonicated intermittently over 48 h with cycles of 3 min sonication and a 7 min interval. The formation of amyloid fibrils was confirmed by TEM.

### Cell culture and PFF transfection

We established a stable SH-SY5Y cell line overexpressing WT human α-synuclein (S3-cell line) and cultured it in a humidified atmosphere of 5% CO_2_ at 37 °C in a 6-well plate in Dulbecco’s modified Eagle’s medium (DMEM) with 4,500 mg/L glucose and L-glutamine, 10% fetal bovine serum (FBS), 50 U/mL penicillin, and 50 μg/mL streptomycin. After reaching confluence, the medium was changed to DMEM with 1% FBS and the cells were transfected with 12.5 μg/mL PFFs or monomers with 3 µg/mL polyethylenimine. After incubation for 24 h, the cells were washed carefully three times with PBS, and the medium was changed to DMEM with 1% FBS without PFFs or monomers. The medium was collected sequentially at 0, 6, and 24 h after medium change, centrifuged at 15,000 × *g* for 30 min, and the supernatant was stored at −80 °C.

### Real-time sonication-induced conversion assay (HANABI assay)

The HANABI system is composed of a microplate reader combined with a water bath-type ultrasonicator, and we used it for a real-time sonication-induced conversion assay. The reaction buffer of the HANABI assay was composed of 500 µg/mL α-synuclein monomers in 1 M NaCl, 50 mM Tris-HCl pH 7.4, and a 10% volume of 100 μM ThT. The total volume of each well was 240 μL. We added a 10% volume of the samples (artificial CSF with PFFs, cell culture medium, or CSF from patients, depending on the experiment) to the reaction buffer. Before they were added to the reaction buffer, the CSF samples were sonicated for 3 min in order to fragment and homogenize α-synuclein aggregates/fibrils and to accelerate the seeding reaction. The reaction mixtures in a 96-well microplate were subjected to cyclic agitation with a 3 min sonication period followed by a 7 min quiescent period. Amyloid formation was monitored by ThT fluorescence (excitation at 450 nm and emission at 485 nm) every 10 min. All samples were measured in 12 replicates, and the time taken to form fibrillary aggregates was evaluated by the average lag time to reach half of the maximum fluorescence in each well. We excluded the wells from the average count in which ThT fluorescence did not increase.

### TEM analysis

Fibril structure was assessed using an H-7650 TEM (Hitachi High Technologies Corporation, Tokyo, Japan) following adsorption onto 400-mesh grids and negative staining with 1% phosphotungstic acid.

### Patients and CSF samples

In August 2015, we launched a prospective observational study of patients with parkinsonism who were admitted to the Department of Neurology, Osaka University Hospital, Osaka, Japan. In this cohort study, we collected clinical data including age, disease duration, drug dose, Hoehn-Yahr scale, MDS-Unified PD Rating Scale (UPDRS) parts I–IV, a detailed series of motor and cognitive function tests, biochemical examination data, magnetic resonance imaging, MIBG myocardial scintigraphy, and dopamine transporter single-photon emission computed tomography (DAT SPECT) using ^123^I-FP-CIT (^123^I-labeled N–ω–fluoropropyl-2β–carbomethoxy-3β-[4-iodophenyl] nortropane). CSF was collected from July 2016 based on the following standardized protocol. CSF was collected by lumbar puncture in the morning after fasting overnight. The CSF samples were centrifuged at 400 × *g* for 10 min at 4 °C, and aliquots were stored at −80 °C.

In the present study, we enrolled all patients with PD who fulfilled the clinical criteria of probable or clinically established PD of the MDS^28^ and received a CSF examination from July 2016 to June 2017. Two specialized neurologists confirmed each diagnosis. Patients with a history of myocardial infarction or thoracic surgery were excluded because of MIBG data analysis. As control patients, we enrolled patients with functional disease, normal pressure hydrocephalus, myopathies, spondylosis, dystonia, myasthenia gravis, and orthostatic hypotension.

### Standard protocol approvals, registrations, and patient consent

This study was approved by the Ethics Committee of Osaka University. Written informed consent was obtained from all patients before enrollment. The study procedures were carried out in accordance with the Declaration of Helsinki.

### Measurement of total α-synuclein levels in CSF

Total α-synuclein levels in CSF were measured using a sandwich ELISA with a human α-synuclein ELISA kit according to the standard protocol (BioLegend, San Diego, CA, USA). All samples were tested in a blinded fashion and in duplicate.

### Measurement of α-synuclein oligomers in CSF by ELISA

Measurement of α-synuclein oligomers in CSF by sandwich ELISA was performed as reported previously with slight modifications^13^. Briefly, an ELISA 96-well plate (Nunc Maxisorb; Thermo Fisher Scientific, Rockford, IL, USA) was coated with 1 µg/mL anti-α-synuclein 211 antibody (Syn211; Thermo Fisher Scientific, Rockford, IL, USA) in 200 mM NaHCO_3_, pH 9.6 (100 µL/well) by overnight incubation at 4 °C, washed with PBS-T, and incubated with 200 µL/well blocking buffer (PBS-T containing 2.5% gelatin) for 2 h. After washing, 100 µL CSF containing 1% protease inhibitor cocktail (Protease Inhibitor Cocktail Set I; Calbiochem, Millipore, San Diego, CA, USA) and 5% heterophilic antibody inhibitor (ELISA diluent; MABTECH, Stockholm, Sweden) was applied to each well and incubated at 37 °C for 3 h, and then incubated with 1 µg/mL biotinylated anti-human α-synuclein 211 monoclonal antibody diluted in blocking buffer at 37 °C for 2 h. The plate was washed and incubated at 37 °C for 1 h with 100 µL/well ExtraAvidin-Peroxidase (Sigma-Aldrich, Dorset, UK). After washing, the plate was incubated with 100 µL/well of an enhanced chemiluminescent substrate (SuperSignal ELISA Femto Maximum Sensitivity Substrate; Thermo Fisher Scientific, Waltham, MA, USA), and the plate was subjected to chemiluminescence measurements with a microplate luminometer (SpectraMax Pro; Molecular Devices Corporation, Tokyo, Japan). The samples were screened in a blinded fashion and tested in triplicate.

### Statistical analysis

Differences in the aggregation kinetics of various concentrations of PFFs were analyzed by one-way analysis of variance (ANOVA). Differences in the kinetics of cell medium with or without PFF seeding were analyzed by two-way ANOVA, using time and conditions as variables. Differences between PD and control patients were analyzed by an unpaired two-tailed t test. Multiple linear regression was performed to predict T1/2 based on age, disease duration, Hoehn-Yahr severity stage, MIBG H/M ratio, and total protein levels of CSF. All statistical analyses were performed with SPSS statistics version 23 (IBM, Armonk, NY, USA). The level of significance was set at p < 0.05.

## Supplementary information

Supplementary information

## Data Availability

The data that support the findings of this study are available from the corresponding author upon reasonable request.
