# Supplementary material for: Ultrasonication-based rapid amplification of α-synuclein aggregates in cerebrospinal fluid
Source: Sci Rep. 2019 Apr 12;9:6001. doi: 10.1038/s41598-019-42399-0 (PMC6461702; doi:10.1038/s41598-019-42399-0)
Supplement: Supplementary file 1 — Supplementary information [file 41598_2019_42399_MOESM1_ESM.pdf]

Title: Ultrasonication-based rapid amplification of α-synuclein aggregates in cerebrospinal fluid.

Authors:
Keita Kakuda[1], Kensuke Ikenaka[1], Katsuya Araki[1], Masatomo So[2], César Aguirre[2], Yuta Kajiyama[1], Kuni Konaka[1], Kentaro Noi[3], Kousuke Baba[1], Hiroshi Tsuda[1], Seiichi Nagano[1], Takuma Ohmichi4, Yoshitaka Nagai[5], Takahiko Tokuda[6], Omar M.A. El-Agnaf[7], Hirotsugu Ogi[3], Yuji Goto[2], Hideki Mochizuki[1]

1. Department of Neurology, Graduate School of Medicine, Osaka University, Yamadaoka 2-2, Suita, Osaka 565-0871, Japan
2. Institute for Protein Research, Osaka University, Yamadaoka 3-2, Suita, Osaka 565-0871, Japan
3. Department of Precision Science and Technology, Graduate School of Engineering, Osaka University, Yamadaoka 2-1, Suita, Osaka 565-0871, Japan
4. Department of Neurology, Kyoto Prefectural University of Medicine, 465 Kajii-cho, Kamikyo-ku, Kyoto 602-8566, Japan
5. Department of Neurotherapeutics, Graduate school of Medicine, Osaka university, Yamadaoka 2-2, Suita, Osaka 565-0871, Japan
6. Department of Molecular Pathobiology of Brain Diseases, Kyoto Prefectural University of Medicine, 465 Kajii-cho, Kamikyo-ku, Kyoto 602-8566, Japan
7. Life Sciences Division, College of Science and Engineering, Hamad Bin Khalifa University (HBKU), Education City, Qatar

*Correspondence to: Hideki Mochizuki. Department of Neurology, Graduate School of Medicine, Osaka University, Yamadaoka 2-2, Suita, Osaka 565-0871, Japan. Email: hmochizuki@neurol.med.osaka-u.ac.jp.

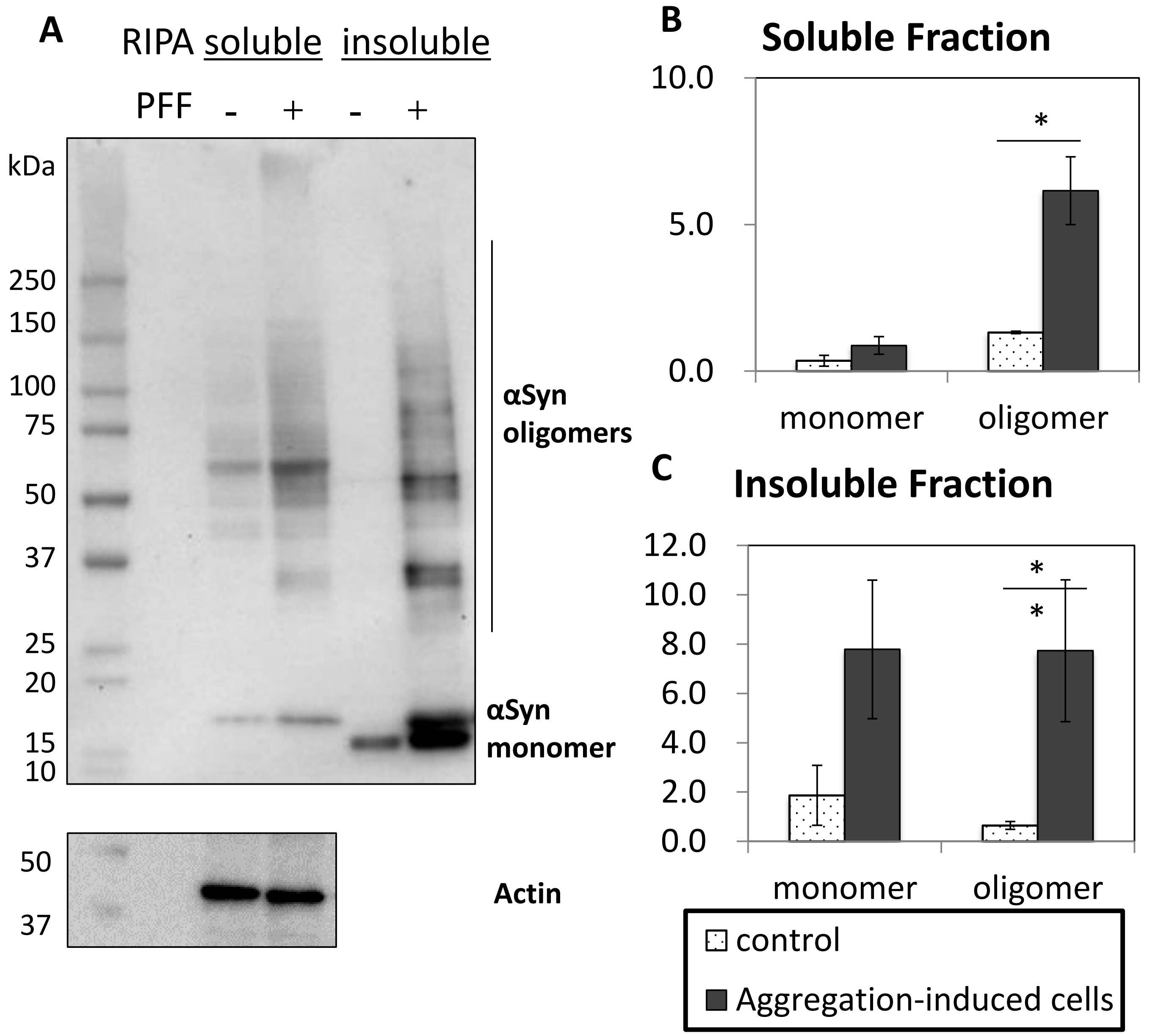

Fig. S1. Intracellular levels of α-synuclein oligomers are elevated in aggregation-induced cells by PFF seeding.

(A) Western blot analysis of the lysates of α-synuclein aggregation-induced cells by PFF seeding and controls. An anti-α-synuclein antibody (Syn 211) was used for detection. (B) Quantitative analysis of the relative amounts of soluble α-synuclein monomers/oligomers adjusted to the levels of actin in the soluble fraction. (C) Quantitative analysis of α-synuclein in the insoluble fraction ($n = 4$). Aggregation-induced cells had higher levels of α-synuclein oligomers than control cells in the soluble and insoluble fractions ($p < 0.01$ and $p < 0.05$, respectively, unpaired two-tailed t-test).

Fig. S2. A representative full-length blot for α-synuclein of cell lysates with the primary Syn 211 antibody. S1-A. The cropped area indicated by the red box is shown in Fig. S1-A.

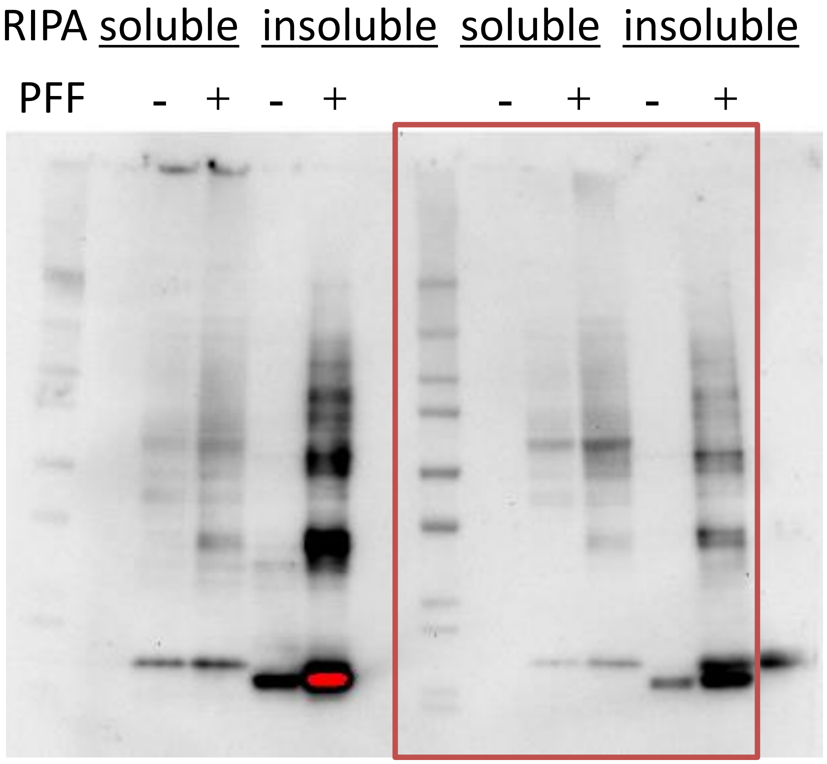

Fig. S3. A representative full-length blot for actin of cell lysates with an anti-actin clone C4 antibody. The cropped area indicated by the red box is shown in Fig. S1-A.

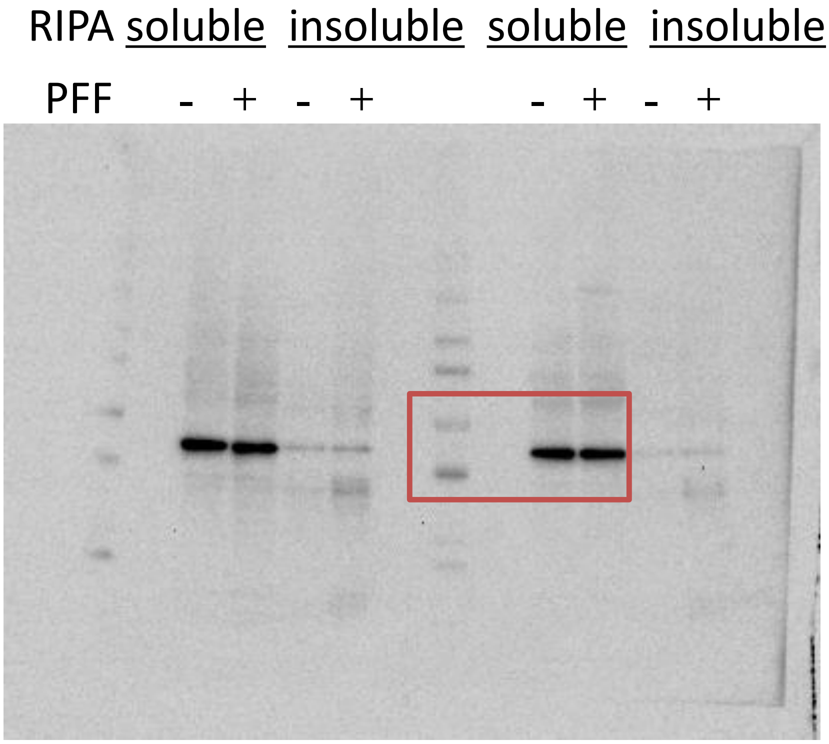

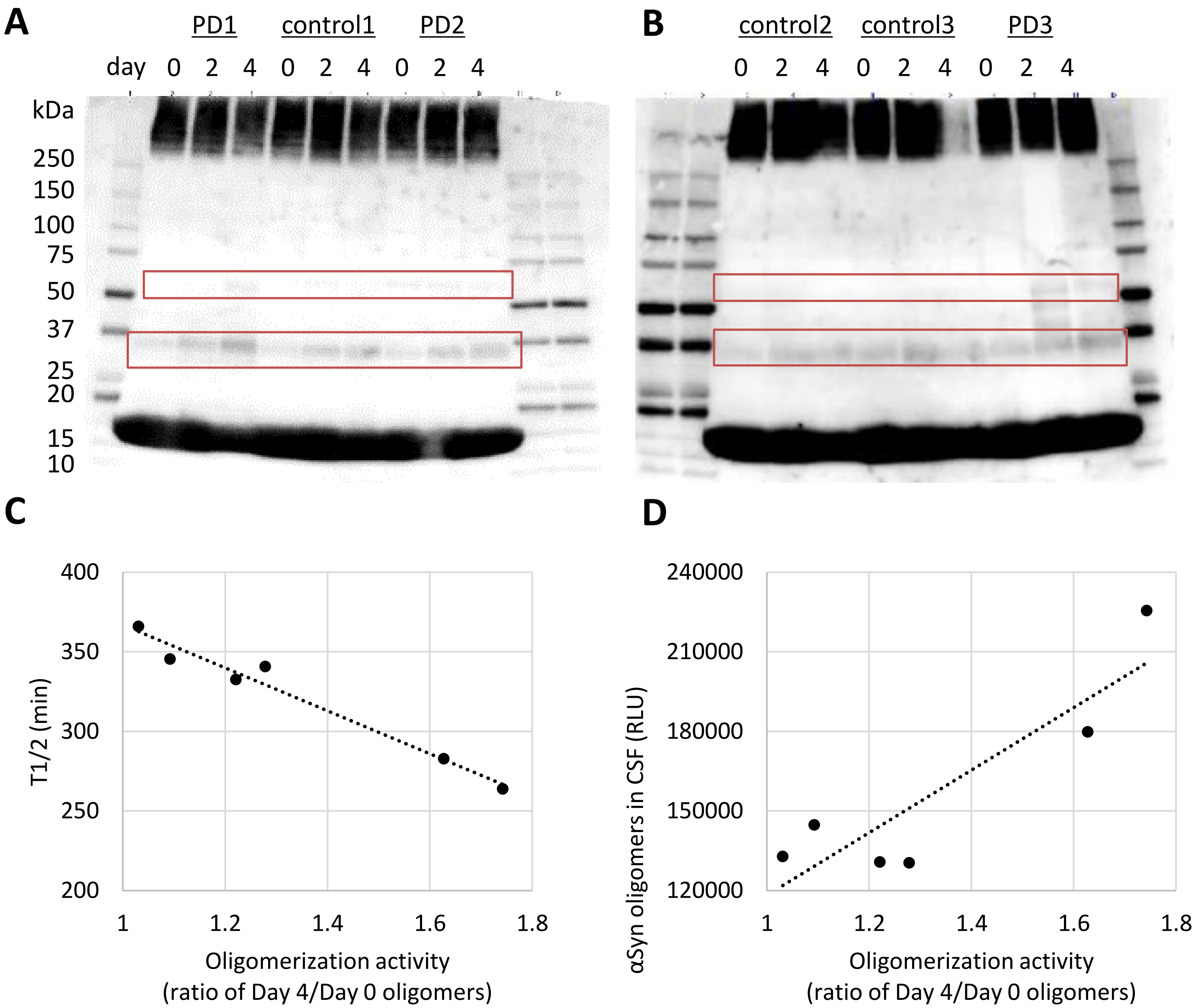

Fig. S4. Oligomeric activity of CSF correlates with the seeding activity of HANABI and α-synuclein oligomers in CSF measured by ELISA.

CSF from six patients (three PD, and three controls) were mixed, respectively, with a pre-filtered α-synuclein monomer solution in 50 mM Tris-HCl and 150 mM NaCl at a v/v ratio of 1:9. Final concentration of α-synuclein was 500 µg/mL. The samples were incubated with continuous shaking (600 rpm) at 37 ° C , and collected at days 0, 2, and 4. The collected samples were centrifuged immediately at 13,500 rpm at 4 ° C for 30 min, and the supernatants were used for the detection of oligomers by western blot analysis. The primary antibody used for western blot was a mouse anti-human α-synuclein oligomer-specific antibody (ASyO5; AS13 2718, Agrisera, Vännäs, Sweden) and the secondary antibody was a sheep anti-mouse peroxidase-linked antibody (NA931; GE Healthcare, Little Chalfont, UK). Oligomerization activity was calculated by the day 4/day 0 ratio of the total intensity of the bands at 33 and 55 kDa.

(A) (B) Western blot analysis of the incubated α-synuclein solution with CSF. The high-molecular band indicated with red boxes was analyzed to calculate the oligomerization activity of each patient. (C) (D) Dot plot of oligomerization of CSF and T1/2 and α-synuclein oligomers in CSF measured by ELISA. Oligomerization activity was significantly correlated with T1/2 (Pearson correlation test, $r = -0.984$, $p < 0.001$) and oligomers in CSF (Pearson correlation test, $r = -0.891$, $p = 0.02$).
